# Circulating exosomes may identify biomarkers for cows at risk for metabolic dysfunction

**DOI:** 10.1038/s41598-019-50244-7

**Published:** 2019-09-25

**Authors:** Fatema B. Almughlliq, Yong Q. Koh, Hassendrini N. Peiris, Kanchan Vaswani, Olivia Holland, Susanne Meier, John R. Roche, Chris R. Burke, Mallory A. Crookenden, Buddhika J. Arachchige, Sarah Reed, Murray D. Mitchell

**Affiliations:** 1 University of Queensland Centre for Clinical Research, Faculty of Medicine, The University of Queensland, Brisbane, Queensland 4029 Australia; 2 Institute of Health and Biomedical Innovation - Centre for Children’s Health Research, Faculty of Health, Queensland University of Technology, Brisbane, Queensland 4101 Australia; 3 DairyNZ Ltd., Private Bag 3221, Hamilton, 3240 New Zealand; 4 Present Address: Ministry for Primary Industries- Manatū Ahu Matua, Pastoral House, Wellington, 6140 New Zealand

**Keywords:** Extracellular signalling molecules, Nanoparticles

## Abstract

Disease susceptibility of dairy cows is greatest during the transition from pregnancy to lactation. Circulating exosomes may provide biomarkers to detect at-risk cows to enhance health and productivity. From 490 cows, animals at high- (n = 20) or low-risk (n = 20) of transition-related diseases were identified using plasma non-esterified fatty acid and β-hydroxybutyrate concentrations and liver triacylglyceride concentrations during the two weeks post-calving. We isolated circulating exosomes from plasma of dairy cows at low-risk (LR-EXO) and high-risk (HR-EXO), and analyzed their proteome profiles to determine markers for metabolic dysfunction. We evaluated the effects of these exosomes on eicosanoid pathway expression by bovine endometrial stromal (bCSC) and epithelial (bEEL) cells. HR-EXO had significantly lower yield of circulating exosomes compared with LR-EXO, and unique proteins were identified in HR-EXO and LR-EXO. Exposure to LR-EXO or HR-EXO differentially regulated eicosanoid gene expression and production in bCSC and bEEL cells. In bCSC, LR-EXO exposure increased PGE_2_ and PGD_2_ production, whereas HR-EXO exposure increased *PTGS2* gene expression. In bEEL, HR-EXO exposure caused a decrease in PGE_2,_ PGF_2α_, PGD_2_, PGFM and TXB_2_ production. The unique presence of serpin A3-7, coiled-coil domain containing 88A and inhibin/activin β A chain in HR-EXO, indicates potential biomarkers for cows at-risk for metabolic diseases. Our results are in line with the health status of the cow indicating a potential diagnostic role for exosomes in enhancing cows’ health and fertility.

## Introduction

The transition from pregnancy to lactation is one of the most challenging periods in a dairy cow’s reproductive cycle. The prevalence of potentially preventable diseases is greatest in the first few weeks after calving, resulting in significant economic losses within dairy industries^1^. During this period, significant metabolic, hormonal, and immunological changes occur, and the body requires an increase in the intake of energy, protein, and minerals^1–3^. When physiological processes fail to successfully coordinate and regulate these metabolic changes, maladaptation can occur, leading to immune dysfunction and increased risk of metabolic and infectious diseases^4–6^. Management programs aim to reduce peripartum diseases, thereby increasing the possibility of an early return to estrus, a successful pregnancy, and improved milk yields^4^. Despite the efforts of applying different nutritional strategies, the occurrence of metabolic diseases and impaired reproductive performance has not declined, and, in fact, may have increased^6^. Biomarkers that can identify pre-pathological diseases have the potential to enhance the effectiveness and profitability of management programs aimed at improving animal health, welfare, and productivity.

In the past decade, new technologies have been utilized to improve diagnostic approaches using exosomes. Exosomes are extracellular vesicles (EVs) 30–120 nm in diameter that are produced by all cell types^7,8^. Exosomes have been detected in biological fluids including plasma, urine, saliva, and milk^9–13^. Through the secretion of exosomes, cells can communicate with adjacent cells, or travel long-distances through the circulatory system to provide tissue-specific messages including proteins, lipids, mRNA, and miRNA^14^. The content of exosomes—and therefore their biological function—is influenced by specific signals from their cell of origin or cues from the cellular milieu^10,15–18^. Their interaction with the targeted cell can directly activate membrane receptors or alter the extracellular environment by releasing their contents into the targeted cell^19^. Circulating exosomes isolated from dairy cows may have potential as indicators of fertility status^20^, uterine diseases^21^, and metabolic diseases^22^, as they carry proteins that are unique to the state of the cow.

Dairy cows have only 12 weeks after calving before needing to reestablish a new pregnancy if they are to maintain a 365-day calving interval, and prostaglandins (PG) play a pivotal role during this period. The general role of endometrial PGF_2α_ is associated with the luteolytic pathway and activation of luteolysis, while endometrial PGE_2_ is key in the luteotrophic pathway and maternal recognition of pregnancy^23^. However, if PG concentrations are altered, this period may also be altered resulting in reduced reproductive performance^24^. We have recently demonstrated that circulating exosomes derived from dairy cows with induced uterine infection decreased the production of PGF_2α_ by endometrial cell lines, showing a direct effect of exosomes on the endometrium’s immune response^25^. However, the effect of exosomes derived from cows that are at-risk for subclinical and clinical diseases on endometrial cells is yet to be discovered.

We have previously investigated exosomal content from transition cows with high and low risk for metabolic diseases^22^ and its effect on kidney cell proliferation^26^; however, these earlier studies were underpowered, with only 5 animals in each group. Here, we greatly expand this research, providing proteome profiles of exosomes derived from the plasma of a larger number of transition cows at high-risk (n = 20) and low-risk (n = 20) for metabolic dysfunction. To investigate the effect of these exosomes on the endometrium, we also evaluated eicosanoid enzyme gene expression and PG production by endometrial cells in an *in vitro* co-incubation model. We hypothesize that during the transition period, circulating exosomes derived from cows at high-risk for metabolic dysfunction contain proteins that are different to those at low-risk. These differing exosomal cargos may be indicative of health, and, therefore, may provide novel biomarkers for disease risk. Additionally, endometrial cells would respond differently to exosomes from high- vs. low-risk animals, demonstrating direct biological effects on reproductive cells.

## Results

### Circulating exosomes derived from cows at low- and high-risk follow the accepted definition of exosomes

**Figure 1:**
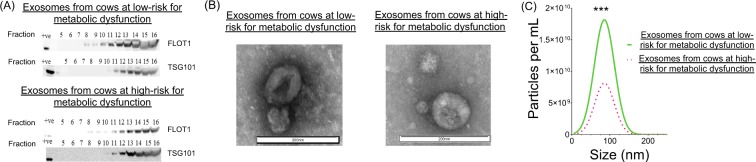
Plasma exosomes from transition cows at low-risk (LR-EXO) and high-risk (HR-EXO) for metabolic dysfunction both confirmed exosomal characteristics and differ in exosome particle numbers. **(A)** Representative western blot for exosomal markers Flotillin 1 (FLOT1) and tumor susceptibility gene 101 (TSG101) of exosomal fractions 5–16 and their presence in exosome-enriched fractions, those exosome-enriched fractions (exosomal samples) were pooled within each animal. **(B)** Spherical shape was confirmed in electron micrographs of exosomes from exosomal samples. **(C)** The size of exosomes (nm) is within the defined size of exosome (30–120 nm), and particle numbers (particle per mL) of exosomal samples was higher in low-risk exosomes. Mann-Whitney test was used to identify the significant differences between groups. ***P ≤ 0.001.

Exosomal fractions isolated from cows at either high- (HR-EXO) or low-risk (LR-EXO) for metabolic dysfunction were both enriched for the exosomal protein markers flotillin 1 (FLOT1) and tumor susceptibility gene 101 (TSG101) (representative immunoblots shown in Fig. 1A), and the vesicles possessed a classical rounded cup shaped morphology (representative TEM images shown in Fig. 1B).

The size of exosomes from LR-EXO and HR-EXO was within the exosome size range and was not significantly different (LR-EXO 85.5 ± 6.07 nm, HR-EXO 85.7 ± 6.68 nm), but exosomal number was reduced (*P* ≤ 0.001) in HR-EXO relative to LR-EXO (fold change 0.78; Fig. 1C).

### Circulating exosomes derived from cows at low- and high-risk had different proteomic profiles

**Figure 2:**
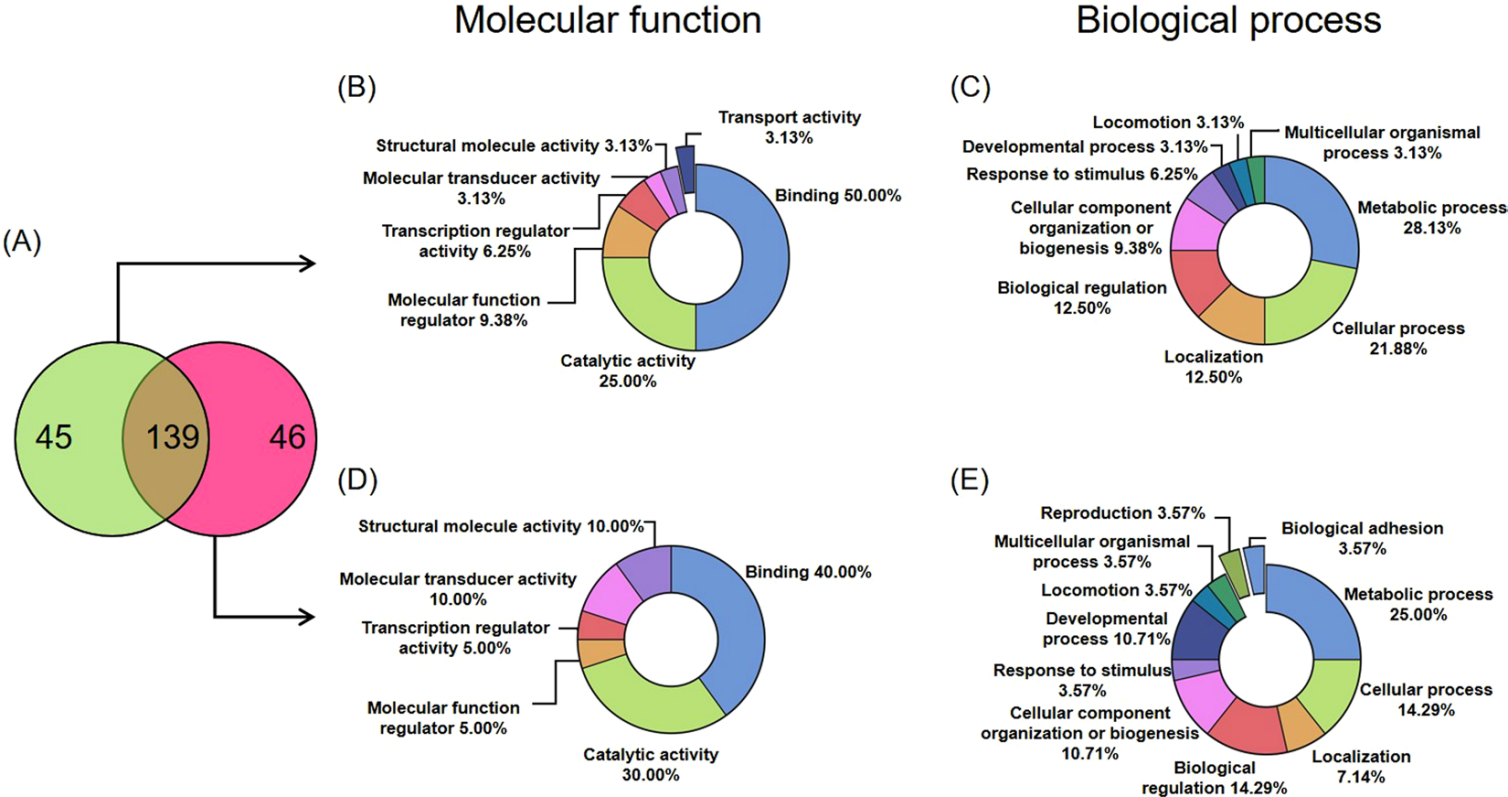
Proteomic analysis reveals that protein content differ between plasma exosomes from transition cows at low-risk (LR-EXO) and high-risk (EXO) for metabolic dysfunction. **(A)** Venn diagram representing unique bovine proteins identifed in LR-EXO (green) or HR-EXO (pink) and common proteins (shaded). Panther analysis revealed differences in bovine proteins from circulating exosomes isolated from LR-EXO cows (**B,C**) and HR-EXO (**D,E**) in gene ontology (GO) classifications.

We identified a total of 184 bovine proteins in LR-EXO and 185 in HR-EXO, with 139 found in both groups, 45 uniquely present in the low-risk group, and 46 uniquely present in the high-risk group (Fig. 2A). Proteins present in both groups predominately had functions in signaling (75.9%), as well as being highly enriched for secreted (51.8%), exosome (49.1%) and microparticle (34.8%) components, and extracellular region (27.7%) (Table 1). A high proportion of proteins were also involved in immune response (12.5 to 25%; Table 1). The shared proteins detected are presented in Supplementary Table 1.

**Table 1:** Functional annotation chart of proteins shared between exosomes isolated from plasma of dairy cows characterized as being at low-risk (n = 20) and high-risk (n = 20) for metabolic diseases during transition period.

Term*	%
Signal	75.9
Secreted	51.8
Extracellular exosome	49.1
Disulfide bond	47.3
Glycoprotein	42.0
Blood microparticle	34.8
Complement and coagulation cascades	28.6
Extracellular space	27.7
Extracellular region	20.5
Negative regulation of endopeptidase activity	15.2
Staphylococcus aureus infection	13.4
Immunity	13.4
Serine-type endopeptidase inhibitor activity	12.5
Innate immunity	12.5
Systemic lupus erythematosus	12.5
Transport	11.6
Protease inhibitor	10.7

*Only terms with a frequency greater than 10% are shown.

The unique proteins were placed into different GO categories based on the PANTHER *Bos taurus* database for LR-EXO (Fig. 2B,C, Supplementary Table 2) and HR-EXO (Fig. 2D,E, Supplementary Table 3). Based on molecular function, PANTHER analysis revealed that most of the exosomal proteins had binding functions (LR-EXO = 50.00%; HR-EXO = 40.00%; Fig. 2B,D) and many had catalytic activity functions (LR-EXO = 25.00%; HR-EXO = 30.00%; Fig. 2B,D). In terms of the biological process categorization, most proteins were related to metabolic process (LR-EXO = 28.13%; HR-EXO = 25.00%) and cellular process (LR-EXO = 21.88%; HR-EXO = 14.29%) (Fig. 2C,E). Nearly double the number of proteins that relate to response to stimulus were detected in LR-EXO (6.25%) compared with HR-EXO (3.57%) (Fig. 2C,E).

### Incubation of circulating exosomes from animals at low- or high-risk of metabolic diseases leads to changes in eicosanoid signaling in stromal and epithelial cells of the endometrium

**Figure 3:**
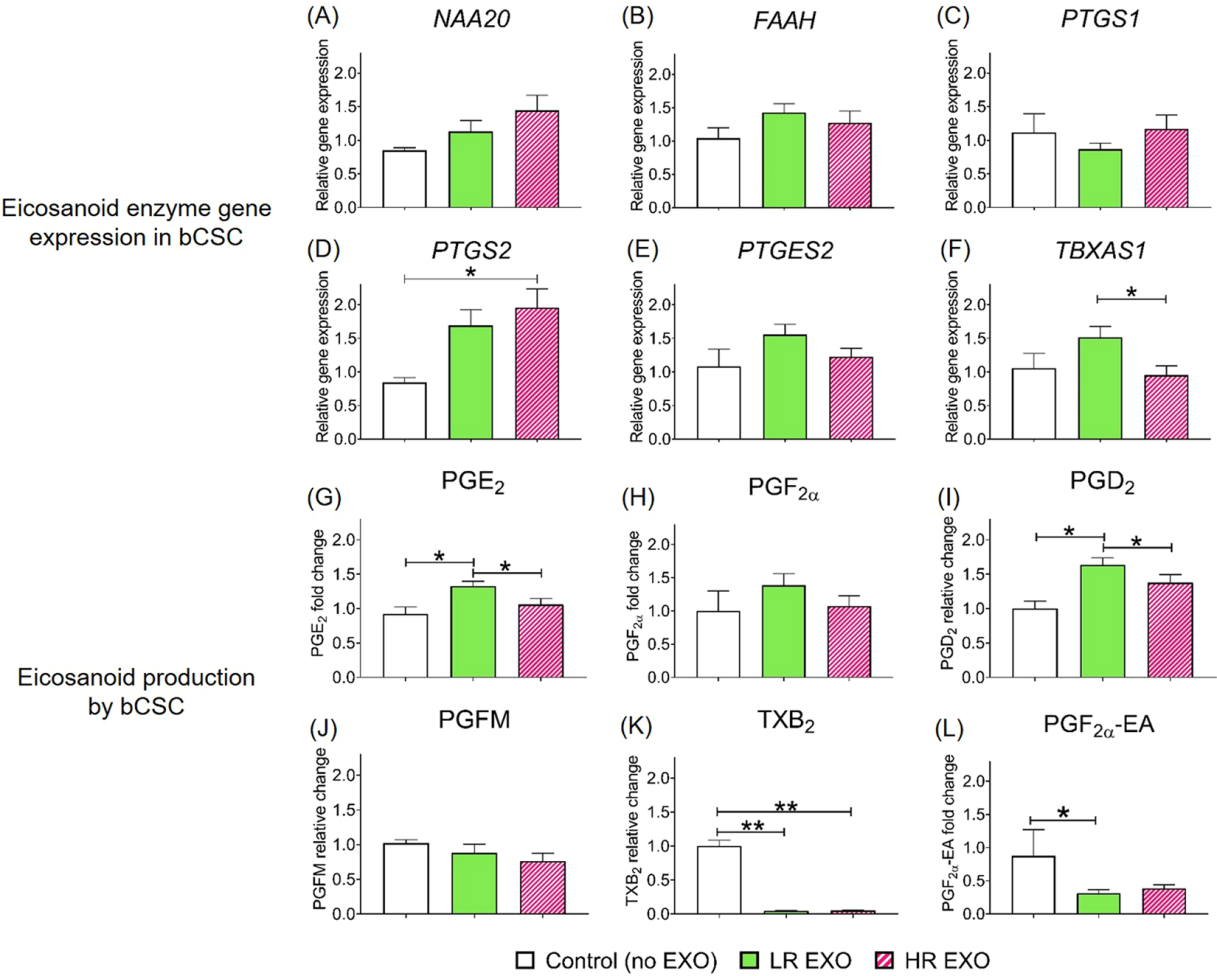
Co-incubation with exosomes from cows at low-risk (LR-EXO) and high–risk (HR-EXO) for metabolic dysfunction lead to changes in eicosanoid expression and secretion in stromal cells. Bovine endometrial stromal cells (bCSC) eicosanoid enzyme gene expressions (**A–F**) and eicosanoid production (**G–L**). Values are presented as mean ± SEM. Mann-Whitney test was used to identify the significant differences between groups. *P ≤ 0.05, **P ≤ 0.01.

**Figure 4:**
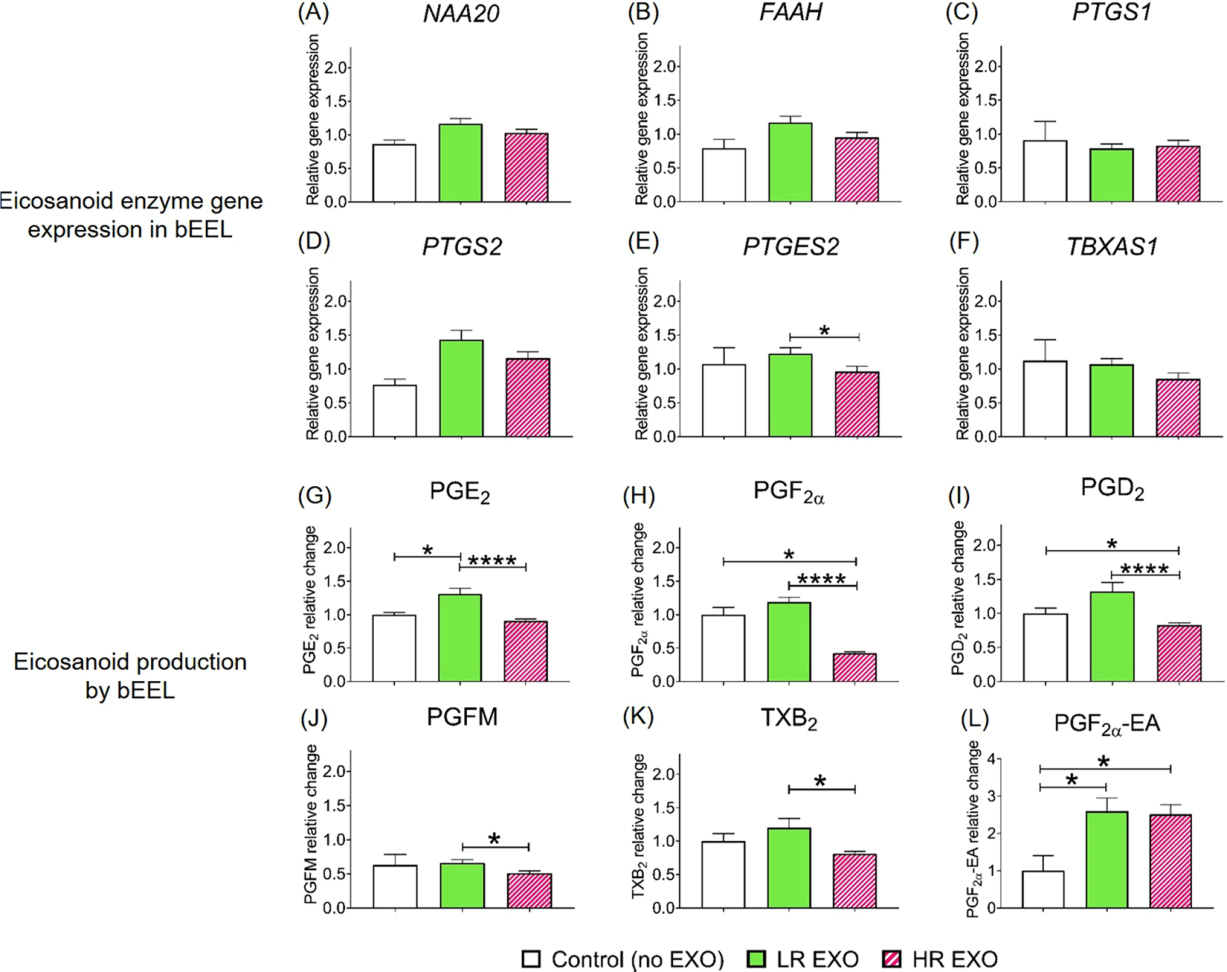
Co-incubation with exosomes from cows low-risk (LR-EXO) and high–risk (HR-EXO) for metabolic dysfunction lead to changes in eicosanoid expression and secretion in epithelial cells. Bovine endometrial epithelial cells (bEEL) eicosanoid enzyme gene expressions (**A–F**) and eicosanoid production (**G–L**). Values are presented as mean ± SEM. Mann-Whitney test was used to identify the significant differences between groups. *P ≤ 0.05, **P ≤ 0.01, ***P ≤ 0.001, ****P ≤ 0.0001.

To investigate the effect of circulating exosomes from low- and high-risk cows on reproductive tissues, bovine endometrial stromal (bCSC) and epithelial (bEEL) cells were exposed *in vitro* to exosomes isolated from each group. Eicosanoid enzyme function in bCSC and bEEL was assessed using real-time PCR (Figs 3A–F and 4A–F, respectively), and eicosanoid production by bCSC and bEEL in the cultured media was measured (Figs 3G–L and 4G–L, respectively).

When bCSC were co-incubated with LR-EXO, no differences were observed in gene expression in comparison to control, but the exposure to HR-EXO resulted in an increase in the gene expression of prostaglandin-endoperoxide synthase 2 (*PTGS2*) and a decrease in the expression of thromboxane A synthase 1 (*TBXAS1*) relative to the control and LR-EXO exposure, respectively (Fig. 3D,F). In terms of eicosanoid production, the co-incubation of LR-EXO with stromal cells resulted in increased production of PGE_2_ and PGD_2_ in comparison to both control and HR-EXO co-incubation (Fig. 3G,I), but thromboxane B_2_ (TXB_2_) and PGF_2α_ ethanolamide (PGF_2α_-EA) production was decreased relative to the control (Fig. 3K,L).

The co-incubation of bEEL with LR-EXO did not alter the gene expression of eicosanoid enzymes, but HR-EXO decreased expression of prostaglandin E synthase 2 *(PTGES2*) in comparison to the co-incubation of LR-EXO (Fig. 4E). The production of PGE_2_ and PGF_2α_-EA was increased when bEEL were exposed to LR-EXO (Fig. 4G,L). HR-EXO resulted in a decrease in the production of PGE_2_, PGFM and TXB_2_ in comparison to LR-EXO, a decrease in the production of PGF_2α_ and PGD_2_ in comparison to control and LR-EXO, and an increase in the production of PGF_2α_-EA in comparison to control (Fig. 4A–F).

## Discussion

Circulating exosomes originate from several different tissues and travel through the blood to targeted tissues or cells, influencing cell behavior. Therefore, circulating exosomes provide a sensitive liquid biopsy of physiological status. In this paper, we provided a comparison of proteins identified in circulating exosomes isolated from dairy cows that were categorized either at high- or low-risk for metabolic diseases during the transition period. The number of circulating exosomes in low-risk cows was greater than in high-risk cows, and exosomal protein content differed between these groups. Additionally, exosomes differentially altered multiple gene expression of eicosanoid enzymes and eicosanoid production by endometrial cells *in vitro*. Our results suggest that exosomes have roles in postpartum dairy cow adaptation to the lactation period and that the adaptive changes in circulating exosomes may provide biomarkers of disease risk.

The research presented here demonstrates improved sensitivity and specificity in exosome isolation compared with previous research conducted in transition dairy cows. Here, we identified 45 and 46 proteins unique to circulating exosomes from cows at low-risk and high-risk, respectively. Previously, fewer than 15 proteins were identified unique to metabolic status of transition cows^22^. The coupling of ultracentrifugation method, to isolate EVs, followed by size exclusion chromatography method, to enrich exosomes, removes most of the overabundant free plasma proteins, thereby minimizing confounding proteins and enabling the identification of unique proteins by mass spectrometry. We confirmed that the exosomes isolated followed the accepted definition of an exosome^27^ by evaluating size (NTA), spherical shape (TEM), and the presence of the exosomal markers TSG101 and FLOT1 (immunoblot). Additionally, our proteomic results confirmed the presence of the plasma-derived exosomal biomarker, CD5 antigen-like (CD5L), in both groups. Common proteins evident in the two groups are involved in intracellular trafficking and vesicular transport, such as sorting nexin-8 (SNX8)^28^ and trafficking protein particle complex subunit (TRAPPC6B)^29^. Further, bioinformatics revealed that the common proteins found in LR-EXO and HR-EXO are largely signal, secreted and exosome proteins. The presence of these proteins from our mass spectrometric results, as well as the exosome characterization results, support the enrichment of exosomes from plasma of transition cows with high- and low-risk for diseases and may provide information for determining biomarkers unique to disease status.

Exosomal proteins unique to the risk groups may be indicative of increased or decreased susceptibility to disease. The total number of proteins detected in the risk groups was similar, 45 proteins were unique to LR-EXO and 46 were unique to HR-EXO; however, when we investigated the proteins that were uniquely present in circulating exosomes from cows with high-risk for metabolic diseases, there were proteins with known functions in disease states.

Peptides identified in circulating exosomes of high-risk cows may be indicative of dampened metabolic health. In the current study, we identified serum amyloid A-4 and serpin A3-7 proteins in the exosomes of transition dairy cows at high-risk of disease. Girolami *et al*. (2018) investigated serum biomarkers of contamination with dioxins and polychlorinated biphenyls **(PCBs**) in dairy cows and identified seven serum proteins associated with contamination. Of note, serum amyloid A-4, serpin A3-7, and fibrinogen β chain demonstrated a progressive decrease in concentration with decreasing contamination^30^. Additionally, serpin A3-7 was previously identified in exosomes derived from dairy cows with uterine infection^21^, and fibrinogen β chain was previously identified in exosomes of high-risk cows one week after calving^22^. Girolami *et al*. (2018) did not describe any other metabolic health parameters, such as non-esterified fatty acids (**NEFA**) or beta-hydroxybutyrate (**BHBA**), which would elucidate the metabolic health status of the contaminated animals. However, contamination with PCBs detrimentally affects (among other tissues and biological processes) the liver, immune system, and reproduction^31^. Collectively, the exosomal protein cargo suggests high-risk animals have overall detrimental health compared with low-risk animals.

Other proteins that were identified in exosomes of high-risk groups are associated with clinical diseases. Here, coiled-coil domain containing 88A (**CCDC88A**) was unique to exosomes of high-risk cows and was previously reported in the kidney cell proteome when co-incubated with exosomes from transition cows at high-risk for metabolic diseases^26^. Coiled-coil interactions are essential in mammalian cells for vesicular trafficking^32^ and vesicle-tethering^33^, which may be one reason why coiled-coil proteins are present in exosomes. However, these proteins have been associated with the fusion machinery of enveloped viral infection^27^. Our results indicate to the possibility that the presence of CCDC88A in the exosomes of high-risk transition cows and their delivery at infectious sites may increase the cow’s susceptibility to infectious diseases.

Inhibin or activin β A chain (**INHBA**) was also unique in exosomes from cows categorized at high-risk of metabolic disease. INHBA is a protein that functions as an inhibitor to secretion of follicle stimulating hormone by the pituitary gland^28^ and has local regulatory roles in various tissues^29^. Increased expression of INHBA was reported in granulosa cells in bovine cystic ovarian disease, which is a critical cause of infertility in dairy cows^34^. The known roles of INHBA in influencing fertility of dairy cows and its unique presence in exosomes from transition cows at high-risk for metabolic dysfunction, may warrant further study of this protein in blood and exosomes as an indicator of infertility.

On the other hand, proteins unique to low-risk cows may be indicative of cows that are at low risk of transition diseases. Toll-like receptor 4 (**TLR4**) was identified uniquely to the low-risk exosomes. TLRs are critical for the innate immune system to recognize pathogen associated molecules and TLR4, in particular, recognizes lipopolysaccharide (**LPS**) from Gram-negative bacteria^35,36^, such as *Escherichia coli*, one of the most common microbes associated with bovine endometrial diseases^37^. The unique presence of TLR4 in exosomes derived from transition cows at low-risk for diseases might indicate that the targeted cells will receive additional immunity compared with cows designated as high-risk of disease and, as such, be able to better defend themselves against uterine infection. The differences between the two groups may reflect, at least to some extent, the role that exosomes might play in protecting cows from the long-term effects of transition period metabolic disease.

Following the proteomic analysis of the circulating exosomes, we examined their effects on eicosanoid expression and production by endometrial stromal and epithelial cell lines. The biosynthesis of eicosanoids is driven by PTGS1 and 2 by converting arachidonic acid (**AA**) to prostaglandin endoperoxide H_2_ (**PGH**_**2**_), which converts rapidly to its metabolites, PGs and TXs, which, together, are called prostanoids. The dominant source of prostanoids is PTGS1, which is constitutively expressed in most cells to maintain homeostasis, but PTGS2 is stimulated in the presence of inflammation to activate eicosanoid pathway^38–40^. However, PTGS2 metabolizes other molecules; anandamide (**AEA**) and 2-arachidonoylglycerol (**2-AG**) to prostaglandin-like products, prostamides (**PMs**) or glycerol esters^41,42^. In our study, endometrial cells expressed *PTGS1* in equal levels in all treatments, which is consistent with its housekeeping function. In endometrial stromal cells, the exposure of HR-EXO increased the expression of *PTGS2*, but did not stimulate production of downstream PGs, which remained at basal level. HR-EXO were derived from cows that are at high-risk for metabolic dysfunction, and the increase in endometrial *PTGS2* expression indicates presence of inflammatory stimuli, which is in line with the health status of cows. LR-EXO also showed a tendency to increase *PTGS2*, but it was not significant.

Endometrial stromal cell production of PGE_2_ and PGD_2_ increased in the presence of LR-EXO signals. Eicosanoids, particularly PGE_2_, have crucial roles in physiological tissue repair and healing, and is always readily responsive to endometrial inflammation and infection^43–47^. This suggests that exosomes in cows at low-risk for metabolic dysfunction contain information that makes eicosanoids readily available to heal tissue damage and to respond to inflammation or infection. In endometrial epithelial cells, high production of PGE_2_ was observed in the presence of LR-EXO; however, HR-EXO caused a more pronounced effect by decreasing PGE_2,_ PGF_2α_, PGD_2_, PGFM and TXB_2_ production. The general pattern of increased eicosanoid production in the presence of LR-EXO and the decrease in the presence of HR-EXO suggests that cows at low-risk may respond faster to inflammation, while the same response in cows at high-risk will be relatively delayed.

A previous study that examined postpartum cows with uterine *Escherichia coli* infection indicated an increase in circulating PGE_2_ but not PGFM, and a switch from PGF_2α_ to PGE_2_ in endometrial explants in response to LPS^43^, which suggests that PGE_2_ is the main PG driver. In our study, endometrial stromal and epithelial cells had similar responses to LR-EXO, but not HR-EXO, where PGE_2_ was increased and PGF_2α_ or PGFM remained unchanged in comparison to basal production. This effect suggests a role for exosomes in a cows’ adaptation to the transition period and quicker recovery responses in cows that are at low-risk for metabolic dysfunction in comparison to those at high-risk.

Although we examined the effects of exosomes on endometrial and epithelial cells, this method is not without limitations. To completely address the roles of exosomes in endometrial eicosanoid interactions during transition period, lipidome analysis of exosome content should be addressed in the future. There is evidence that exosomes can carry eicosanoids or compounds related to their pathways such as phospholipases, arachidonic acid, PGs, leukotrienes, regardless of the species and their involvement in reproduction^48–51^. Not only PGs were carried by exosomes but also PTGS1 and 2, demonstrating that exosomes can be a production site for eicosanoids^48^. Therefore, the lipid content of the exosome could influence the cellular responses of endometrial cell lines after the incubation of the exosomes from the two groups.

Circulatory exosomes can be used to identify potential biomarkers at risk of metabolic disease. Here, we described exosomal cargo that is indicative of decreased liver function, immune function, and overall health in high-risk animals, and a possible link with fertility status. The identification of exosomal proteins, such as Serpin A3-7, CCDC88A and INHBA may be harbingers of additional factors that exacerbate infectious disease and metabolic dysfunction, which have long-been associated with reproductive failure. Overall, the unique proteins identified in cows divergent in metabolic health, indicate the potential for exosomal cargo to be used as biomarkers of health status in transition dairy cows. The current study is a basic discovery approach for the identification of potential biomarkers for cows at risk; however, validation cohorts of the potential candidate proteins through a targeted quantification approach would lead to the development of those proteins as markers. A thorough investigation into the functions of exosomal cargo on eicosanoid production by endometrial cells, may reveal key biological signals that would aid the assessment of health and fertility status of dairy cows. The changes in exosomal content identified here may provide novel targets for the future development of diagnostic biomarkers of metabolic health in cattle; however, additional research will be required to determine if biomarkers can perform better than current methods. We aim to deliver robust and replicable findings to the point at which scalability becomes possible. Further investment may take our initial discoveries to a commercial test.

## Materials and Methods

### Animals

Holstein-Friesian primiparous cows used in this study were part of a larger experiment (approved by the Ruakura Animal Ethics Committee AEC#13574, #14200) and were managed in a pasture-based, spring-calving dairy system. All experiments were performed in accordance with relevant guidelines and regulations.

From the larger group of calved cows (n = 490), 40 cows were identified as being at either high-risk (n = 20) or low-risk (n = 20) of metabolic dysfunction. This was based on plasma BHBA (mmol/L), plasma NEFA (mmol/L), and liver triacylglycerol (**TAG**, % of liver wet weight) concentrations within the first two weeks of calving. Risk category of individual cows was based on their metabolic health status 7 and 14 days (plasma BHBA and NEFA) and 10 days (liver TAG) after calving (Table 2). The remaining 450 animals with intermediate risk of metabolic disease were not investigated further.

**Table 2:** Categorization of transition dairy cows into high-risk (n = 20) and low-risk (n = 20) for metabolic dysfunction groups using known indicators of metabolic health.

Indicator	High-risk	Low-risk	*P-value*
Plasma BHBA^a^ day 7 (mmol/L)	1.434 ± 0.096	0.525 ± 0.027	≤0.0001
Plasma BHBA day 14 (mmol/L)	2.169 ± 0.234	0.645 ± 0.034	≤0.0001
Plasma NEFA^b^ day 7 (mmol/L)	1.245 ± 0.102	0.645 ± 0.038	≤0.0001
Plasma NEFA day 14 (mmol/L)	1.250 ± 0.092	0.605 ± 0.054	≤0.0001
Liver TAG^c^ day 10 (mg/wet weight)	3.802 ± 0.406	1.333 ± 0.035	≤0.0001

^a^Beta-hydroxybutyrate. ^b^Non-esterified fatty acids. ^c^Triacylglycerol.

Blood samples for metabolite analysis (day 7 and day 14) and exosome extraction (day 10) were collected by coccygeal venipuncture into evacuated blood tubes containing either heparin or EDTA anticoagulant, respectively. Blood was placed immediately on ice and centrifuged at 1, 500 × *g* for 12 min at 4 °C; then, the plasma was aspirated and stored at −20 °C until analysis. Approximately 1 g (wet weight) of liver tissue was collected, on average, 10 days (SD 0.98 d) post-calving for TAG analyses as previously outlined by Roche *et al*.^52^. The collected liver samples were snap-frozen in liquid nitrogen and stored at −80 °C until analyses. Gribbles Veterinary Pathology Laboratory undertook the analyses; plasma BHBA in Hamilton (NZ) and plasma NEFA and liver TAG in Dunedin (NZ), respectively. Plasma BHBA was assayed with a Roche reagent kit using colorimetric technique on a Hitachi Modular P800 analyses at 37 °C (Roche Diagnostics), plasma NEFA concentration were measured using a Wako Chemicals kit (Osaka Japan), and liver TAG content analyzed using a Wako LabAssay Triglyceride kit (290–63701, Wako Chemicals USA Inc, USA).

### Extracellular vesicle isolation from plasma by ultracentrifugation

Extracellular vesicles (**EVs**) were isolated from a total of 40 plasma samples by successive differential centrifugation steps, conducted as previously described^53^. Briefly, the plasma was centrifuged (4 °C) at 2,000 × *g* for 30 min and then 12,000 × *g* for 30 min. The supernatant was filtered through a 0.22-μm filter, and centrifuged at 100,000 × *g* for 2 h at 4 °C using a fixed angle ultracentrifuge rotor Type 70.1 Ti (Beckman Coulter, California, USA). The 100,000 × *g* pellet (EVs) was reconstituted in 500 µL PBS (Gibco, Thermo Fisher Scientific Australia Pty Ltd, Scoresby Vic) and stored at −80 °C.

### Exosome separation and purification by size exclusion columns (SEC)

Extracellular vesicles obtained from ultracentrifugation were loaded onto qEV size exclusion columns (Izon, Oxford, UK) for exosome isolation, as per manufacturer instructions. This technique allows the separation of particles from EV pellet based on their size into 16 fractions. The 16 fractions were concentrated using a vacuum centrifuge for 1.5 h at room temperature. Plasma exosomes of each animal were stored at −80 °C for exosome characterization and further analysis.

### Protein quantification

Quantification of protein concentration of exosomal fractions was evaluated using Bicinchoninic Acid (**BCA**) assay (Sigma-Aldrich, St Louis, MO, USA) and bovine serum albumin (Sigma-Aldrich, St Louis, MO, USA) dilution was used as standards.

### Immunoblotting

Immunoblotting followed the same method done by Almughlliq, *et al*.^25^ to identify the exosomal fractions, which were then pooled accordingly. Exosomes (10 µg protein) were further characterized using gel electrophoresis (NuPAGE Novex 4–12% Bis-Tris, Thermo Fisher Scientific Australia Pty Ltd, Scoresby Vic) for confirmation of exosomal markers Flotillin 1 (**FLOT1**) and tumor susceptibility gene 101 (**TSG101**). The gel was then transferred to a PVDF membrane (Bio-Rad Laboratories Pty., Ltd, Australia) using Trans-Blot Turbo system (Bio-Rad Laboratories Pty., Ltd, Australia). After blocking (5% skim milk powder), membranes were probed overnight with primary antibodies anti-FLOT1 (1:500; Abcam, Cambridge, United Kingdom) and, anti-TSG101 (1:500; Abcam, Cambridge, United Kingdom) at 4 °C, followed by secondary anti-rabbit IgG (1:1000 Sigma–Aldrich, St Louis, MO, USA) and secondary anti-goat (1:1000; Sigma–Aldrich, St Louis, MO, USA), respectively. Membranes were then covered with SuperSignal West Dura-Extended Duration Substrate (Thermo Fisher Scientific Australia Pty Ltd, Scoresby Vic). Targeted proteins were visualized on X-ray films using Konica SRX101A processor (Konica Minolta medical and graphic INC, Japan).

### Transmission electron microscopy

Transmission electron microscopy was used to visualize exosome particles from exosomal fractions. Exosomes (5 μL) were added onto formvar coated copper grids for 2 min, then briefly washed in ultrapure water and negatively stained with 1% uranyl acetate. The samples were than visualized using the JEOL 1010 transmission electron microscope operated at 80 kV, and images captured using an Olympus Soft Imaging Veleta digital camera^53^.

### Nanoparticle tracking analysis

Based on the presence of exosomal markers, exosomal fractions 10–16 were pooled. The size distribution and total exosome particle number (particles/mL) of each plasma exosome sample were determined using the nanoparticle tracking instrument (NanoSight NTA 3.1 Nanoparticle Tracking and Analysis Release Version Build 0064) as previously described^10^.

### Proteomic analysis of plasma-derived exosomes by tandem mass spectrometry

Proteomic was processed and analysed following the method done by Almughlliq, *et al*.^21^. Each plasma exosome sample (10 μg of protein) was sonicated to expose proteins from the inside of the exosome. Samples were then treated with 50 mM ammonium bicarbonate and 20 mM dithiothreitol and reduced for 1 h at 65 °C. For alkylation, samples were then incubated with 100 mM iodoacetamide in the dark for 1 h at 37 °C, followed by an overnight incubation with trypsin at a 1:50 ratio (Sigma–Aldrich, Castle Hill, NSW, Australia) at 37 °C. The following day, 0.1% formic acid in H_2_O was added to each sample to stop the trypsin reaction and to acidify the samples. Samples were then desalted according to a modified version of the stage tip protocol^54^. Briefly, a 3-mm piece of an Empore C18 (Octadecyl) SPE Extraction Disk was excised and placed in a gel loader tip and POROS slurry (5 μL) was added to form a microcolumn. Trifluoroacetic acid (1 volume, 0.1% in water) was added to the sample and loaded onto the microcolumn. The microcolumn was washed with trifluoroacetic acid (20 μL, 0.1% in water). Peptides were eluted from the microcolumn by three washes of acetonitrile (20 μL × 3, 0.1% formic acid). The digested protein samples were dried in a vacuum centrifuge (Eppendorf Concentrator Plus, NSW, Australia) before spectral acquisition. Samples were reconstituted in formic acid (50 μL, 0.1%), vortexed for 10 sec and centrifuged for 2 min at 10, 000 × *g* to remove particulates^10,54–56^.

Protein samples were then analyzed using the TripleTOF® 5600 mass spectrometer (ABSciex, Redwood City, CA) coupled to an Eksigent nano ultra 1D + HPLC system. The ChromXP C18-CL TRAP (10 mm × 0.3 μm, 120 Å) and analytical ChromXP C18 columns (0.075 µm × 150 mm; 3 µm, 120 Å) (Eksigent, Redwood City, CA) were used to separate the digested proteins. A 5 μL aliquot of digested material was injected onto the column and separated with a linear gradient of 2% to 40% Buffer B for 60 min (Buffer A: 0.1% Formic acid/water; Buffer B: acetonitrile/0.1% formic acid), 40 to 65% Buffer B (5 min), 65 to 95% Buffer B (5 min), with a flow rate of 300 nL/min. The column was flushed with 95% Buffer B for 9 min and re-equilibrated with 2% Buffer B for 10 min. The in-depth proteomic analysis was performed using the Information Dependent Acquisition experiments on the TripleTOF® 5600 System interfaced with a nanospray ion source. The source parameters were as follows: Curtain gas value was 25 (arbitrary units), ion source gas 10 and declustering potential 70 (arbitrary units). A 250 msec accumulation time was set for the TOFMS survey scan and from this scan, the 12 most intense precursor ions were selected automatically for the MS/MS analysis (accumulation time of 150 msecs per MS/MS scan).

MS results were analyzed using the ProteinPilot™ Software (v5.0 beta, AB Sciex, Redwood City, CA), with the Paragon algorithm against UniProt bovine species database (http://www.uniprot.org/). False discovery rate (**FDR**) was estimated using a reversed sequence database. For a discovery approach, all samples were run separately then protein calls from found uniquely in each group (low or high risk) or shared between groups were combined into three data sets for *in silico* analysis. Three or more peptides were required to positively identify a protein as present. To further analyze the proteins identified by MS, PANTHER (**PANTHER**; http://www.pantherdb.org/) was used to place those proteins into different gene ontology (**GO**) categories on the basis of the PANTHER *Bos taurus* database^57^. The data were processed and categorized based on “biological process” and “molecular function” with the ontology and pathway analysis using the “protein analysis through evolutionary relationships” tool.

### Endometrial cell culture

Bovine endometrial stromal (**bCSC**) and epithelial (**bEEL**) cell lines were kindly gifted by (Université Laval, Québec)^58^. They were grown in RPMI media (Gibco, Thermo Fisher Scientific Australia Pty Ltd, Scoresby Vic) containing exosome depleted 10% fetal bovine serum (Bovorgen, Interpath Services Pty Ltd, Australia), 1000 U/mL antibiotic-antimycotic solution (Gibco, Thermo Fisher Scientific Australia Pty Ltd, Scoresby Vic) and incubated at 37 °C and 5% CO_2_^59^. Experiments were conducted in media without fetal bovine serum.

### Functional studies of exosomes on endometrial cells

For cell culture, bCSC (seeding density of 8,000 cells per well) and bEEL (seeding density of 35,000 cells per well) were incubated for 24 h grown in RPMI media (Gibco, Thermo Fisher Scientific Australia Pty Ltd, Scoresby Vic) containing 10% fetal bovine serum (Bovorgen, Interpath Services Pty Ltd, Australia) and 1000 U/mL antibiotic-antimycotic solution (Gibco, Thermo Fisher Scientific Australia Pty Ltd, Scoresby Vic). For the co-incubation experiment (treatment with exosomes), FBS free RPMI media, containing 1000 U/mL antibiotic-antimycotic solution (Gibco, Thermo Fisher Scientific Australia Pty Ltd, Scoresby Vic) was used^25^. Cells were incubated with RPMI media with no addition of exosomes (No EXO control, for baseline measurements), or treated with HR-EXO (n = 20) or LR-EXO (n = 20) with 1 × 10^8^ particles per well for 24 h to analyze gene expression and in separate wells at 36 h to measure eicosanoid expression in culture media at 37 °C and 5% CO_2_. We performed 2 well replicates per individual cow (n = 40). Exosome concentration for co-incubation was chosen in reference to literature^60^ and incubation time from Srinivasan *et al*.^61^. Cell culture experiments were performed in duplicate per cell line. Cells and cultured media was collected and stored at −80 °C until required for further analyses.

### Eicosanoid enzymes gene expression analysis

Gene expression was analyzed after 24 h of LR-EXO and HR-EXO treatments on bEEL and bCSC. Following Almughlliq, *et al*.^62^, the bovine custom RT^2^ Profiler PCR Array (CAPA9696-24; Qiagen, VIC, Australia) was used to quantify the gene expression of eicosanoid enzymes (*NAA20* (PPB11826A)*, FAAH* (PPB11713A)*, PTGS1* (PPB00825A), *PTGS2* (PPB00826A)*, PTGES2* (PPB01910A) and *TBXAS1* (PPB04022A)) by reverse-transcriptase quantitative PCR. The PCR arrays were cycled using the following parameters: 1 × (10 min at 95 °C) followed by 40 × (15 sec at 95 °C and 1 min at 60 °C) cycles using the QuantStudio ™ 3 Real-Time PCR System (Applied Biosystems ™, Foster City, CA). The endogenous control genes included on the array were TATA box binding protein (*TBP*) and hypoxanthine phosphoribosyltransferase 1 (*HPRT1*). Real-time PCR specificity was confirmed by analyzing the melting curves. PCR reproducibility, reverse transcription efficiency and the presence of genomic DNA contamination were verified before analyzing further. Housekeeper genes were not changed with treatment. Gene expression results were normalized to the endogenous control genes *HPRT1* and *TBP*. Real-time PCR data were analyzed using comparative *C*_T_ method^26,63^.

### Eicosanoid extraction from cell culture media

Eicosanoid metabolites were extracted from cell culture media for each sample using the following method described in Mitchell *et al*.^64^. Briefly, extraction solution was made up using 99% methanol (Sigma-Aldrich, Australia) and 1% of 0.1% formic acid in water containing 50 μL (250 fmol) of each internal standard (IS; 1 μM); for prostaglandin E_2_-d4 (PGE_2_-d4), prostaglandin F_2α_-d4 (PGF_2α_-d4), PGD_2_-d4, 13,14-dihydro-15-keto-PGF(2alpha) -d4 (PGFM-d4), prostaglandin E_2_-ethanolamide-d4 (PGE_2_-EA-d4), and prostaglandin F_2α_-ethanolamide-d4 (PGF_2α_-EA-d4) (Cayman chemicals, USA). In a 96-well extraction plate (Phenomenex, USA), 100 μL of each sample/standard and 250 µL of extraction solution was added and vortexed (900 rpm, 5 min) followed by addition of 750 μL chilled water, place Teflon mat on, and vortexed (900 rpm, 5 min). A Solid Phase Extraction plate (SPE; Phenomenex Australia Pty Ltd) was pre-equilibrated consecutively using 500 μL methanol (Sigma-Aldrich, Australia) and 2 × 500 μL water (Sigma-Aldrich, Australia) via the use of a vacuum manifold. The prepared standards and samples were loaded on to the SPE plate and left to bind for 1 min. The SPE plate was then washed twice with chilled water via the use of a vacuum manifold vacuum (<5 mm Hg). The bound lipid extract preparation was eluted by loading 500 μL acetonitrile and dried using a vacuum concentrator (overnight at room temperature). The dried lipids were then reconstituted in 20% methanol and prepared in triplicate (sample replicates).

### Eicosanoid measurements using liquid chromatography tandem mass spectrometry (LC-MS/MS)

For separation of the sample analytes, we used UPLC (Shimadzu, Japan) coupled with a Kinetek C8 column (Phenomenex, USA) attached to a guard column (Phenomenex, USA). Oven temperature was set to 60 °C and 15 min gradient was set up using aqueous and organic mobile phase solvent preparations. A multiple reaction monitoring (MRM) method was carried out for the analyses and quantitation of 6 analytes (PGE_2_, PGF_2α_, PGD_2_, PGFM, TXB_2_ and PGF_2α_-EA,) and the deuterated versions (PGE_2_-d4, PGD_2_-d4, PGF_2α_-d4, PGFM-d4, and PGF_2α_-EA-d4) using negative and positive modes (Table 3). 20 uL of each sample was injected into the mass spectrometer (QTRAP 5500 LCMS system, SCIEX, USA)^64^.

**Table 3:** Optimized multiple reaction monitoring (MRM) method pairs and parameters for eicosanoids.

Endogenous lipids	Q1 (Positive)	Q3 (Positive)	Q1 (Negative)	Q3 (Negative)
PGE_2_			351.1	271.1
PGF_2α_			353.2	309.0
PGD_2_			351.0	271.3
PGFM			353.2	113.0
TXB_2_			369.0	169.1
PGF_2α_-EA	380.4	62.0		
**Internal standards**	**Q1 (Positive)**	**Q3 (Positive)**	**Q1 (Negative)**	**Q3 (Negative)**
PGE_2_-d4			355.1	319.0
PGF_2α_-d4			357.2	313.0
PGD_2_-d4			355	193.2
PGFM				
PGF_2α_-EA-d4	384.4	62.0		

MultiQuant Software (SCIEX, USA) was used to analyze individual peaks for each analyte. Peaks were selected using +/−0.5 min retention time window. The MultiQuant data was used to generate area and peak area ratio (PAR) information for both standards and samples. Standard curves were plotted with individual concentrations against the PARs, and the line equation was used to extrapolate the concentrations (pg/mL) for the samples.

### Statistical analysis

Mann-Whitney test was used to identify the statistically significant differences between control and each treatment. Prism Software (prism7, GraphPad Inc., La Jolla, CA) was used for the statistical test. Data are presented as sample means ± SEM. Statistical significance was defined as *P* < 0.05.

## Supplementary information

Supplementary Information: The full unedited blots used in Fig. 1A of exosomal fractions 5–16. The cropped blots presented in Fig. 1A are shown in the boxes.

Supplementary Dataset 1

## Data Availability

All data needed to evaluate the conclusion in the paper are present in the paper.
