# Supplementary material for: Circulating exosomes may identify biomarkers for cows at risk for metabolic dysfunction
Source: Sci Rep. 2019 Sep 25;9:13879. doi: 10.1038/s41598-019-50244-7 (PMC6761115; doi:10.1038/s41598-019-50244-7)
Supplement: Supplementary file 1 — Supplementary Information: The full unedited blots used in Fig. 1A of exosomal fractions 5–16. The cropped blots presented in Fig. 1A are shown in the boxes. [file 41598_2019_50244_MOESM1_ESM.pdf]

Circulating exosomes may identify biomarkers for cows at risk for metabolic dysfunction

Fatema B. Almughlliq, Yong Q. Koh, Hassendrini N. Peiris, Kanchan Vaswani, Olivia Holland, Susanne Meier, John R. Roche, Chris R. Burke, Mallory A. Crookenden, Buddhika J. Arachchige, Sarah Reed and Murray D. Mitchell.

Exosomes from cows at low-risk for metabolic dysfunction

TSG101 (45 kDa)

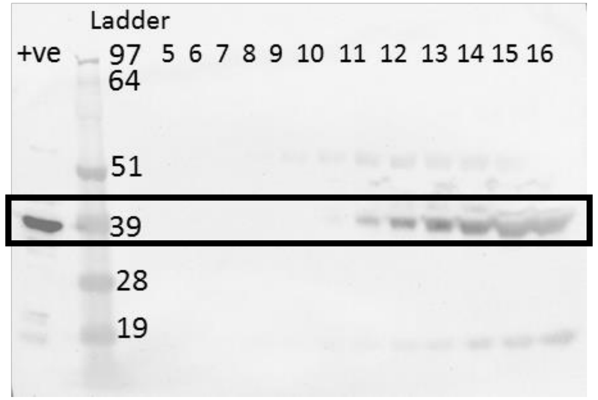

FLOT-1 (49 kDa)

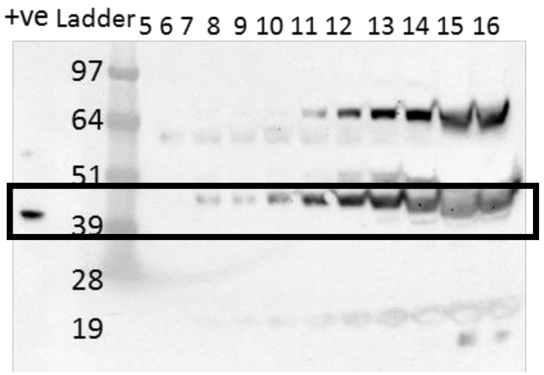

Exosomes from cows at high-risk for metabolic dysfunction

TSG101 (45 kDa)

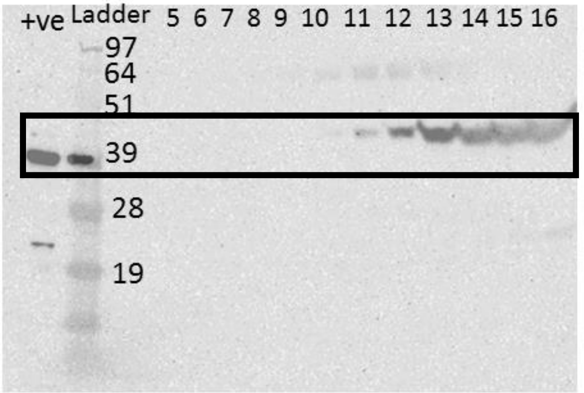

FLOT-1 (49 kDa)

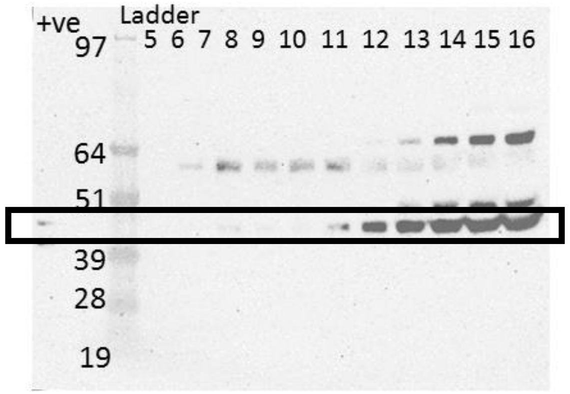

Supplementary Information: The full unedited blots used in Fig. 1A of exosomal fractions 5–16. The cropped blots presented in Fig. 1A are shown in the boxes.
